# Feasibility, reliability and validity of smartphone administered cognitive ecological momentary assessments in breast cancer survivors

**DOI:** 10.3389/fdgth.2025.1543846

**Published:** 2025-04-22

**Authors:** Ashley M. Henneghan, Emily W. Paolillo, Kathleen M. Van Dyk, Oscar Y. Franco-Rocha, Mansi Patel, So Hyeon Bang, Raeanne C. Moore

**Affiliations:** ^1^ School of Nursing, University of Texas at Austin, Austin, TX, United States; ^2^ Dell Medical School, Department of Oncology, University of Texas at Austin, Austin, TX, United States; ^3^ Department of Neurology, Memory and Aging Center, University of California, San Francisco, Weill Institute for Neurosciences, San Francisco, CA, United States; ^4^ Semel Institute of Neuroscience and Human Behavior, University of California Los Angeles, Los Angeles, CA, United States; ^5^ Jonsson Comprehensive Cancer Center, University of California Los Angeles, Los Angeles, CA, United States; ^6^ Department of Neuroscience, College of Natural Sciences, University of Texas at Austin, Austin, TX, United States; ^7^ School of Nursing, Columbia University, New York, NY, United States; ^8^ UC San Diego Health Sciences, University of California San Diego, San Diego, CA, United States

**Keywords:** ecological momentary assessments, mobile cognitive testing, cancer-related cognitive impairment, breast cancer survivors, reliability, validity, feasibility

## Abstract

**Objective:**

Breast cancer and its treatment are associated with cancer-related cognitive impairments (CRCI). Cognitive ecological momentary assessments (EMA) allow for the assessment of individual subjective and objective cognitive functioning in real world environments and can be easily administered via smartphones. The objective of this study was to establish the feasibility, reliability, and validity of a cognitive EMA platform, NeuroUX, for assessing CRCI in breast cancer survivors.

**Methods:**

Using a prospective design, clinical cognitive assessments (neuropsychological testing; patient reported outcomes) were collected at baseline, followed by an 8-week EMA smartphone protocol assessing self-reported cognitive concerns and objective cognitive performance via mobile cognitive tests once per day, every other day. Satisfaction and feedback questions were included in follow-up data collection. Feasibility data were analyzed using descriptive methods. Test–retest reliability was examined using intraclass correlation coefficients for each cognitive EMA (tests and self-report questions), and Pearson's correlation was used to evaluate convergent validity between cognitive EMAs and baseline clinical cognitive variables.

**Results:**

105 breast cancer survivors completed the EMA protocol with high adherence (87.3%) and high satisfaction (mean 87%). Intraclass correlation coefficients for all cognitive EMAs were strong (>0.73) and correlational findings indicated moderately strong convergent validity (|0.23| < *r* < |0.61|).

**Conclusion:**

Fully remote, self-administered cognitive testing for 8-weeks on smartphones was feasible in breast cancer survivors who completed adjuvant treatment and the specific cognitive EMAs (cognitive EMA tests and self-report questions) administered demonstrate strong reliability and validity for CRCI.

## 1 Introduction

Cancer-related cognitive impairments (CRCI), which present as difficulties with attention, memory, processing speed, and executive functioning, can occur during and/or after breast cancer treatment. Estimates vary throughout the cancer continuum and by measurement methods (i.e., cognitive testing vs. self-report), but approximately 30%–78% of breast cancer patients and survivors experience CRCI (1, 2) which can significantly reduce quality of life and daily functioning (3, 4). Both formal neuropsychological evaluation of objective cognitive functioning across cognitive domains and valid and reliable self-report instruments are recommended to measure CRCI (5, 6). These assessment methods; however, are somewhat limited. For example, neuropsychological testing only captures cognitive performance at a snapshot in time, traditional self-report measures can be influenced by retrospective recall and state-dependent (e.g., mood) biases, and both methods may not be sensitive enough to detect subtle changes in cognitive function in this population (7).

Cognitive ecological momentary assessments (EMAs) are increasingly being used to address these limitations with traditional clinical measures and capture cognitive performance in natural environments (8). Other advantages of cognitive EMAs include greater accessibility, allowing users to take assessments remotely while enabling frequent monitoring to track cognitive changes over time. They also improve ecological validity by capturing data in real-world settings, reduce costs, enhance engagement through user-friendly interfaces, and can integrate passive data collection for deeper insights into cognition-in-context. Cognitive EMAs have demonstrated sensitivity to cognitive changes in adults with mild cognitive impairment (9, 10), and have recently been applied to study CRCI (11–13). In newly diagnosed breast cancer patients (*n* = 49), an 89% adherence rate was observed for those that completed an EMA protocol (5 separate bursts of 7-day EMAs across 4 months). However, only 55% of the participants enrolled completed the full study, and the authors did not report data on the psychometric properties of the EMA (11). Another group demonstrated the preliminary feasibility, reliability, and validity of delivering a 14-day (5 times/day) cognitive EMA protocol to breast cancer survivors (*n* = 47), and reported that EMAs may be more sensitive to CRCI than traditional assessments (12, 13). However, the sample was limited to stage I and II breast cancer survivors.

Other research questions related to CRCI, that can last months to years after treatment (1, 2), may require a longer assessment period (i.e., >2 weeks) for EMA protocols than have been evaluated to-date. To our knowledge, no studies have evaluated the psychometric quality of cognitive EMAs in breast cancer survivors for >2 weeks, which is critical first step to applying these methods to this population and addressing the aforementioned gaps in knowledge. Our team recently demonstrated the feasibility, reliability, and validity of a commercially available cognitive EMA platform (NeuroUX, Inc.) for assessing cognitive functioning (mobile cognitive test performance and self-report questions) in a sample of women living with metastatic breast cancer (*n* = 51) once per day for 4 weeks (28 sessions) (14).

In this brief report, we build on previous work (11–13, 15) and evaluate the feasibility, reliability, and validity of an 8-week cognitive EMA protocol, including mobile tests and self-report questions, once every other day (28 sessions total) to assess cognitive functioning in a sample of non-metastatic (0–III) breast cancer survivors who completed their primary breast cancer treatment (i.e., surgery, chemotherapy, or radiation therapy). The rationale for administering cognitive EMAs for this period of time (i.e., every other day for 8 weeks) is to provide psychometric data to inform future studies that need to monitor cognitive function for longer periods of time, such as behavioral interventions for CRCI [which are commonly delivered over multiple weeks/months (16)]. In the present observational study, we do not expect to find general trends of improvement/decline across participants, rather participant specific fluctuations across the 8 weeks. The objectives of this study were to: (1) describe feasibility metrics (adherence, satisfaction, utility), and (2) evaluate psychometric characteristics (within-person variability, reliability, and convergent validity) metrics of eight ecological mobile cognitive tests and two self-reported cognitive EMAs in a sample of breast cancer survivors.

## 2 Methods

### 2.1 Design

An intensive longitudinal (prospective observational) design was used. All study-related procedures were conducted in accordance with the Declaration of Helsinki and approved by the University of Texas at Austin Institutional Review Board (STUDY00002393).

### 2.2 Sample

We enrolled women who were at least 21 years old, lived in the U.S., had been diagnosed with and completed primary treatment for breast cancer (stage 0–III) within the previous 6 years. Study procedures were conducted remotely from the University of Texas at Austin. We recruited nationally through breast cancer social networks (e.g., the Breast Cancer Resource Center, breastcancer.org, Keep A Breast) and the UCLA Clinical and Translational Science Institute cancer registry.

### 2.3 Procedures

Participants provided informed consent. All participants completed clinical assessments of cognitive function at baseline via Research Electronic Data Capture (REDCap) surveys hosted at the University of Texas at Austin (17, 18) and remote administration of a cognitive test battery via BrainCheck (19). Cognitive EMAs were administered across 8-weeks via NeuroUX (once daily every other day for 8 weeks; 28 assessments total participant). Post-study feedback surveys were administered following EMA protocol via REDCap to assess satisfaction and utility. The detailed protocol, including overall design, sampling procedures, inclusion/exclusion criteria, recruitment, and enrollment for this study has been previously described (7, 15). Key methodologic details for these analyses are as follows.

### 2.4 Clinical cognitive assessments

Baseline REDCap surveys included questionnaires to capture sociodemographic characteristics (e.g., age, education, race, ethnicity, marital status, children/dependents, income, employment), health history (e.g., co-morbidities, menstrual history, current medications) and cancer history (e.g., breast cancer type/stage, cancer treatment details, end date of chemotherapy) and the Functional Assessment of Cancer Treatment Cognitive Function version 3 (“FACT-Cog”) to assess self-reported cognitive function (20). The perceived cognitive impairments subscale (20 item) was used for convergent validity analyses. A computerized battery of standardized neuropsychological tests were administered at baseline via BrainCheck (BrainCheck, Inc.) to assess objective cognitive performance, including: the Trail Making Tests for attention and processing speed, the Digit Symbol Substitution Test and the Stroop Test for executive functioning, and the Recall Test (list learning) for immediate and delayed verbal memory (19). Raw scores were used in convergent validity analyses (i.e., median time between clicks for Trails A and B, Stroop median reaction times, Digit Symbol median reaction time between clicks for all trials and number correct per second, number of correct responses for immediate and delayed memory, and the raw combined score).

### 2.5 EMA protocol details

Weblinks were texted to participants at varied times of day throughout the study period and remained active for 6 h. Reminder texts were sent after 3 h and after 5 h, if assessments were not completed. Each assessment took approximately 10 min to complete and included two Likert-type scale (responses could range from 0 to 7) rating for cognitive symptoms (i.e., “how bad are cancer-related cognitive symptoms”, higher indicates worse symptoms) and confidence in cognitive abilities (i.e., “how confident are you in your cognitive abilities”, higher indicates more confidence) followed by four mobile cognitive tests which tapped into cognitive domains of working memory, executive functioning, processing speed, and memory. Cognitive tests alternated between two different tests per cognitive domain throughout the protocol (see Supplementary Table 1 for full testing protocol). To assess working memory, we used the N-Back (using a 2-back design, 12 trials each test) and the CopyKat tests. The CopyKat is similar to the popular electronic game Simon, where participants are presented with a 2 × 2 matrix of colored tiles in a fixed position. The tiles briefly light up in a random order, and participants are asked to replicate the pattern by pressing on the colored tiles in the correct order. N-back and CopyKat scores were used in these analyses.

For executive functioning, we used the Color Trick and the Hand Swype tests. Color Trick asks the participant to match the color of the word with its meaning for 15 trials (total score and median reaction time on Color Trick were used in analyses). Hand Swype asks participants to swipe in the direction of a hand symbol or in the direction that matches the way the symbols are moving across the screen, with instructions switching throughout the task (Hand Swype reaction time and scores were used in these analyses). For processing speed, we used the Matching Pair and Quick Tap 1 tests. In Matching Pair, the participant is asked to quickly identify the matching pair of tiles out of 6 or more tiles. Matching Pair is a time-based task and runs for 90 s (total score was used in the analyses). For Quick Tap 1, participants are asked to wait and tap the symbol when it is displayed (12 trials/administration; median reaction time were used in the analyses).

For visual/spatial memory we used the Variable List Memory Test and Memory Matrix tests. For the Variable List Memory Test, participants are provided with a list of 12 or 18 random words and given 30 s to memorize the list. Then they are asked yes/no questions to determine if words were on the list or not, immediately following the memorization time (total words correct was used for analyses). For Memory Matrix, patterns are quickly displayed to the participant, then they are asked to indicate the pattern that was displayed by touching the tiles that were in the pattern. This test gets progressively harder if responses are correct (total score was used in the analyses). These specific NeuroUX ecological mobile cognitive tests (i.e., CopyKat; Color Trick; Hand Swype; Matching Pair; Quick Tap 1; Variable List Memory Test; Memory Matrix) have demonstrated acceptable to strong reliability and validity in previous studies of community dwelling adults, women living with metastatic breast cancer, and adults with mild cognitive impairment (14, 21, 22).

### 2.6 Data analyses

For feasibility, descriptive statistics were calculated for adherence rates (number of partial/completed cognitive EMAs across the 28 administrations). To assess acceptability of cognitive EMAs, the following questions were asked:

1. “Overall, how satisfied are you with your experience participating in this study?” Responses ranged: 0–100 (0 = unsatisfied, 50 = neutral, and 100 = very satisfied).
2. “How challenging was it for you to answer the survey questions and do the brain games on your smartphone during the protocol?” Responses ranged: 0–100 (0 = not challenging, 50 = neutral, and 100 = very challenging).
3. “Would you be open to incorporating smartphone based cognitive tasks as part of your ongoing care to monitor your cognitive functioning?” Responses were “Yes”, or “No”.

Consistent with previously published methods (9, 21, 23, 24), including our cognitive EMA protocol in women living with metastatic breast cancer (15), NeuroUX data were cleaned to remove instances of suspected low effort/engagement (see Supplementary Table 2 for cleaning and outlier removal rules). NeuroUX raw scores were then transformed into z scores (mean = 0, SD = 1).

Test–retest reliability was examined for each mobile cognitive test by calculating intraclass correlation coefficients (ICC) using the ICC (2,k) model/type (25). Pearson correlations evaluated convergent validity between gold-standard clinical cognitive measures collected at baseline (FACT Cog (20); raw BrainCheck cognitive test scores for Trail Making Tests, Digit Symbol Substitution Test, Stroop Test, and Recall Test (19) and raw scores for cognitive EMA measures (person-specific mean cognitive EMA performance and EMA self-report cognitive symptoms). Linear mixed effects models were used to evaluate practice effects of the cognitive EMAs (raw scores). Linear and quadratic effects of time (defined as study day) on each test score were examined, and person-specific random intercepts and effects of time were modeled. For tests scores with significant quadratic practice effects, mixed effects models with linear splines tested whether there was a timepoint where improvements in performance level off. Since time of day of EMA administration varied for each participant throughout the protocol, the relationship between the time of assessment and performance on cognitive tests was explored using linear mixed effects models with person-specific intercepts.

Scatterplots between all specified variables were examined for linearity, boxplots were examined for presence of any remaining outliers, and scatterplots of residuals for all associations tested were examined for homoscedasticity. All assumptions were met. False Discovery Rate (FDR) *p*-value adjustment was applied to correct for multiple comparisons. All analyses were conducted in R version 4.3.2.

## 3 Results

Between May 2023 and July 2024, 111 women enrolled in the study and completed baseline data collection, of those 105 initiated the cognitive EMA protocol (reflecting an accrual rate of 94.6%). Women that initiated the cognitive EMA protocol were on average 51.2 years old (SD: 12.0) The sample was approximately 2.1 (SD: 1.6) years post adjuvant treatment completion. See Table 1 for demographic and clinical characteristics.

**Table 1:** Demographic and clinical characteristics of the sample (*N* = 105).

Demographic characteristic	Mean (SD), median	Frequency (percentage)
Age	51.2 (12), 49	—
Age groups		
Ages 24–39	—	19 (%)
Ages 40–60	—	59 (%)
Ages 61–88	—	27 (%)
Race/Ethnicity		
White	—	73 (69.5%)
Asian/Asian American	—	10 (9.5%)
Black/African American	—	9 (8.6%)
Hispanic	—	11 (10.5%)
Other or prefer not to say		2 (1.9%)
Employment		
Working Full or Part Time	—	70 (66.7%)
Unemployed due to disability/laid off	—	9 (8.6%)
Retired	—	17 (16.2%)
Full time homemaker	—	5 (4.8%)
Prefer not to answer	—	4 (3.8%)
Years of education	17.3 (2.9), 17	—
Clinical Characteristic	Mean (SD), median	Frequency (Percentage)
Years since end of adjuvant treatment	2.1 (1.6), 2.0	—
Time since treatment groups		
<1 year	—	33 (31.4%)
1–3 years	—	41 (39.1%)
>3 years	—	31 (29.5%)
Breast Cancer Stage		
0	—	9 (8.6%)
I	—	36 (34.3%)
II	—	37 (35.2%)
III	—	16 (15.2%)
Unsure stage, nonmetastatic	—	7 (6.7%)
Treatments History		
Tumor removal surgery	—	103 (98.1%)
Radiation	—	82 (78.1%)
Hormonal Treatment	—	64 (61%)
Chemotherapy	—	71 (67.6%)
Targeted Treatment	—	36 (34.3%)
Additional reconstructive surgery	—	46 (43.8%)
Received Neoadjuvant chemotherapy	—	37 (35.2%)
Currently on hormone treatment	—	63 (60%)
Participants with 1 comorbidity	—	27 (25.7%)
Pre-menopausal	—	17 (16.2%)
Peri-menopausal	—	12 (11.4%)
Post-menopausal	—	65 (61.9%)
Other, unknown menopausal status	—	11 (10.5%)

Adherence rates for the cognitive EMA protocol were on average 87.3% (SD = 13.2%) and ranged from 41%–100% across participants, so we explored if low adherence early in the protocol (defined as in week 1) was predictive of low adherence throughout the 8-week protocol. We found a large correlation between adherence rate in the first 7 days and overall adherence (*r* = 0.73, *p* < .0001, see Supplementary Figure 1). Eleven of the participants (10.5%) had adherence rates below 70%. We explored sociodemographic and clinical differences (as described in Table 1) between those with low adherence compared to high adherence (using independent samples *t*-tests and Chi square tests) and found none. Baseline cognitive tests scores, FACT-Cog PCI scores, and cognitive EMA scores (person-specific means) were also compared between the low/high adherence groups. Overall, those with low (<70%) adherence self-reported significantly worse cognitive symptoms (both with EMA and baseline assessments) but performed comparably on objective cognitive testing (both mobile and baseline) compared to those with high adherence (see Supplementary Table 3).

Participants expressed high satisfaction with the study protocol, with an average satisfaction rating of 87 out of 100 (SD: 15.1, median 90.5, range 50–100). For perceived challenge of the EMA protocol, average ratings suggested an overall “neutral” level of challenge though there was a wide range (mean rating: 45.2, SD: 28.3, median: 50, range: 0–91). The low adherence group rated the EMA protocol as significantly more challenging than the high adherence group [67.43 (20.99) compared to 43.25 (28.06), *t* = −2.21, *p* = 0.029]. In response to the question about incorporating smartphone based cognitive tasks as part of ongoing care to monitor cognitive functioning, 76.4% of the sample responded “yes” and 16.8% responded “maybe”.

**Figure 1:**
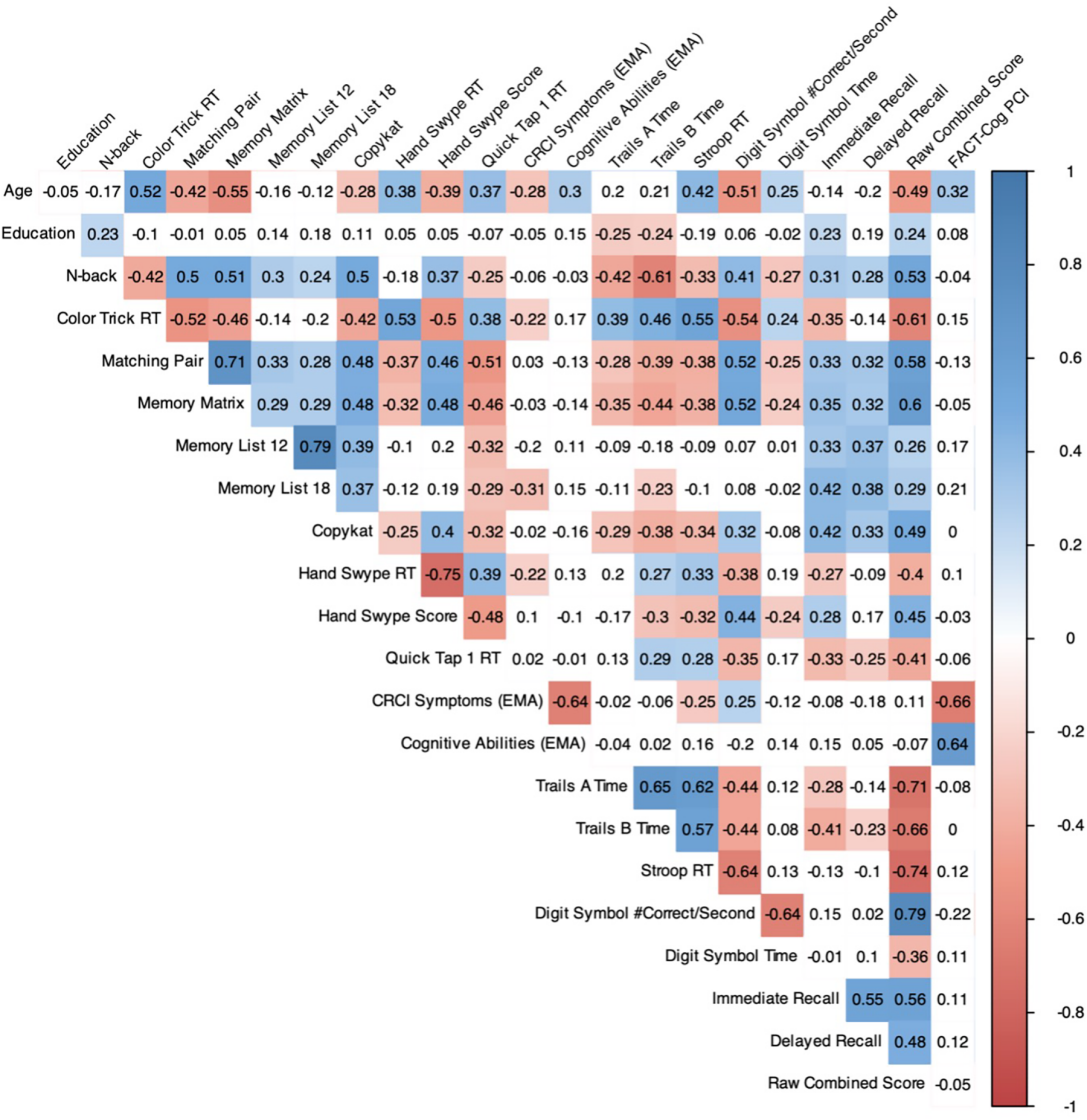
Pearson correlation matrix of cognitive EMAs and clinical assessments of cognitive function (*n* = 105). Colors represent the strength of the correlation only for statistically significant associations (FDR-corrected). EMA, ecological momentary assessment; FACT-Cog PCI, Functional Assessment of Cancer Treatment Cognitive Function perceived cognitive impairment scale.

Average score distributions for each of the cognitive EMAs and within-person variability metrics (average within-person SD) are displayed in Table 2. All cognitive tests demonstrated moderate to excellent (26) test–retest reliability (ICC's > 0.729). Correlations among EMA cognitive tests (mean performance/rating) and age, years of education, BrainCheck raw test scores, and self-reported cognitive function (FACT-Cog PCI) are shown in Figure 1 (95% CIs for all correlation coefficients can be found in Supplementary Table 3). All cognitive EMA test scores showed expected relationships with age (higher age = worse performance), though age associations with Memory List 12 and 18 did not reach statistical significance. Only N-back had a significant association with education (*r* = 0.23, *p* = 0.021).

**Table 2:** Cognitive EMA score distributions, within-person variability, and reliabilities (*n* = 105).

Cognitive EMAs	Average score distribution		Within-person variability		Test–Retest reliability
	Mean (SD)	Range	Average Within-Person SD	Range of Within-Person SDs	ICCs [95%CI]
N-back score	6.16 (1.52)	2–8	1.25	0–2.62	0.95 [0.93,0.96]
Color Trick median reaction time	2,225.64 (754.56)	1,171.54–5,745.25	642.09	131.69–2,026.56	0.94 [0.92,0.95]
Matching Pair score	309.53 (59.26)	196.18–462.92	51.57	20.66–83.87	0.94 [0.92,0.95]
Memory Matrix score	42.76 (7.64)	28.14–64.5	7.44	2.57–13.62	0.92 [0.90,0.94]
Memory List 12 score	21.86 (1.5)	17.11–24	1.46	0–4.26	0.86 [0.81,0.90]
Memory List 18 score	30.96 (2.53)	23.6–34.75	2.28	0–7.07	0.73 [0.64,0.80]
CopyKat score	11.54 (2.8)	6.69–23.5	3.22	1.32–7.62	0.89 [0.86,0.92]
Hand Swype median reaction time	1,832.86 (454.33)	1,180.36–3,398.39	358.79	105.08–1,103.17	0.96 [0.95,0.97]
Hand Swype score	27.8 (7.11)	7.33–44.1	5.67	1.58–12.11	0.97 [0.96,0.97]
Quick Tap 1 median reaction time	404.9 (77.82)	277.75–759.08	50.81	9–167.96	0.96 [0.95,0.97]
Cognitive symptoms	1.65 (1.57)	0–6.65	0.7	0–2.08	0.99 [0.98,0.99]
Cognitive abilities	4.37 (1.45)	0.91–7	0.91	0–2.77	0.98 [0.97,0.98]

ICC, intraclass correlation coefficients; SD, standard deviation.

Cognitive symptoms were assessed with 1 item Likert type question, “I have cancer-related cognitive, or brain, symptoms” and responses from 0 to 7, with 0 indicating not at all, and 7 indicating extremely. Cognitive abilities were assessed with a 1 item Likert type question, “I am confident in my cognitive abilities (thinking, memory, concentration)” and responses from 0 to 7, with 0 indicating not at all, and 7 indicating extremely.

All cognitive EMA test scores also showed significant medium-to-strong associations in expected directions with the BrainCheck global score (“Raw Combined Total”). Within domains, mean verbal memory performance was most strongly associated with BrainCheck memory scores, including Immediate Recall (Memory List 12: *r* = 0.33, *p* < 0.001; Memory List 18: *r* = 0.42, *p* < 0.001) and Delayed Recall scores (Memory List 12: *r* = 0.37, *p* < 0.001; Memory List 18: *r* = 0.38, *p* < 0.001). Working memory performance (CopyKat) also showed a medium-strength association with BrainCheck Immediate Memory (*r* = 0.42, *p* < 0.001) in addition to its significant associations with BrainCheck speed and executive measures. N-back, another working memory test, had a notably strong relationship with BrainCheck Trails B time (*r* = −0.61, *p* < 0.001). Cognitive EMA tests of processing speed (i.e., Matching Pair, Quick Tap) were significantly associated with nearly all BrainCheck scores. Cognitive EMA tests of executive functioning (i.e., Color Trick, Memory Matrix, Hand Swype) were also significantly associated with all or nearly all BrainCheck tests. Divergent validity was also evident, as cognitive EMAs assessing verbal memory showed no significant relationships with BrainCheck non-memory tasks (*p*'s > 0.05).

Self-reported cognitive EMAs were most strongly correlated with baseline self-reported cognitive functioning on the FACT-Cog PCI. Notably, greater EMA-self-reported CRCI symptoms were unexpectedly associated with faster BrainCheck Stroop reaction times (*r* = −0.25, *p* = 0.043) and better BrainCheck Digit Symbol performance (*r* = 0.25, *p* = 0.037). Given the significant negative association between age and EMA-self-reported CRCI symptoms and BrainCheck Stroop and Digit Symbol performance, we re-ran the analysis after controlling for age, and found that the associations of CRCI Symptoms with BrainCheck Stroop (*b* = −0.041, SE = 0.027, *p* = 0.127) and Digit Symbol (*b* = 0.007, SE = 0.006, *p* = 0.207) were no longer statistically significant (see Supplementary Table 5 for full model results). In contrast, EMA-reported CRCI Symptoms showed an expected negative association with Memory List 18 (*r* = −0.31, *p* = 0.004) that held even when covarying for age (*b* = −0.597, SE = 0.158, *p* < 0.001).

Overall significant non-linear practice effects (i.e., a quadratic effect of time) were observed for the majority of the cognitive EMAs. Linear practice effects were observed for Memory List 18 whereas no consistent practice effects (linear or non-linear) were observed for Memory 12 or CopyKat. Linear splines revealed that for all cognitive EMAs with non-linear practice effects, improvements in performance leveled off between the 9th study day and 19th study day, depending on the test. For additional detail, see all practice effects results in Supplementary Figure 2. For time of day of EMA administration, linear mixed effects models revealed reaction times appeared to be slower later in the day compared to the morning, while memory appeared to be slightly better mid-day and evening compared to morning (see Supplementary Table 6).

## 4 Discussion

We found that cognitive EMA monitoring across 2 months is feasible in breast cancer survivors as shown by high accrual rates, overall excellent adherence to the protocol, and high satisfaction neutral challenge ratings. The specific cognitive EMAs (cognitive EMA tests and self-report questions) demonstrated strong reliability and convergent validity with gold-standard measures to assess CRCI. Our overall adherence rate of 87.3% is higher than general EMA protocol adherence rates reported in meta-analyses (range from 75% to 80%) (27). The high adherence and satisfaction scores we found may be attributed to specific study related procedures used to minimize burden such as factoring participant preferences into the timing of EMA texts each day, sending reminders, troubleshooting technical difficulties, and providing instructional support for the specific mobile cognitive tests. Some of the participants demonstrated lower adherence rates to the protocol, which were unlikely due to sociodemographic or clinical characteristics, but may have been a function of lower perceived cognitive functioning at baseline and/or greater perceived challenge of completing the study EMA protocol. aken together, findings demonstrate that breast cancer survivors are overall interested and engaged in ongoing monitoring of their cognitive functioning via smartphone technologies.

The specific cognitive EMA protocol, including mobile cognitive tests and self-rated symptom assessments, administered via NeuroUX, demonstrated strong reliability across administrations and very good convergent validity overall, which is consistent with strong validity and reliability of different assessment items and mobile cognitive tests administered across 14 days in early-stage breast cancer survivors (13). The lowest reliability was found for Memory List tests—12-item (0.86) and 18-item (0.73), which is not only consistent with our recent reports of reliability metrics of memory list 12 and 18 scores in women living with metastatic breast cancer (15), but also expected since these tests generate new and unrelated lists of words with each administration.

Our findings are also comparable to previously published NeuroUX data (21). While this study did not include a control group, our findings can be compared to results from a large sample of cognitively unimpaired adults living in the U.S. for these same mobile cognitive tests of memory (Memory Lists, Memory Matrix), reaction time (Matching Pair, Quick Tap 1), and executive functioning (Color Trick, CopyKat, Hand Swype) (21). Our sample demonstrated similar scores and variability in memory tests, but slightly slower reaction times on Quick Tap 1 and Hand Swype tests. Greater within-person variability in specific cognitive domains (i.e., processing speed) has been found in older adults with mild cognitive impairment compared to those without impairment, suggesting mobile cognitive assessments can differentiate between people with and without cognitive impairment (10). Slowed thinking and “brain fog” is often reported by cancer survivors, so it is possible that reaction time on cognitive EMAs may be sensitive to CRCI. The predictability of within-person variability in cognitive EMAs for development of cognitive impairment should be explored in future research.

Adequate convergent validity was found for these cognitive EMAs supported by medium-to-strong correlations among baseline clinical cognitive assessments, with strongest correlations identified for tests of specific cognitive domains (e.g., tests of memory correlating with BrainCheck memory) and self-reported cognitive functioning. Age and education correlated with the objective cognitive EMAs in expected directions, but education was not significantly related with any. This is expected as NeuroUX tests were designed to be minimally influenced by socioeconomic status factors, including educational attainment, to provide equitable assessment of cognitive function across diverse populations (28).

Significant correlations also emerged in this sample among EMA for CRCI symptoms and objective cognitive tests (both EMA and BrainCheck), but not between the standard clinical cognitive assessments of subjective (FACT-Cog) and objective (BrainCheck) function (see Figure 1). However, the directionality of these relationships suggests that survivor's self-reported CRCI may be most reflective of memory-related symptoms after controlling for age. However, the EMA item for CRCI symptoms in this study was not domain specific, so we cannot further test this hypothesis. Future EMA research for CRCI should include self-report items for different cognitive domains. It is also possible that the relationship between perceived cognitive function and objective cognitive performance may be age-dependent, where younger survivors may face more cognitive demands in their daily lives and/or are more aware of cognitive failures than older survivors who may either not experience as many cognitive demands or expect cognitive symptoms as a function of “older age”. Future studies should focus on disentangling the moderating effects of age on the relationship between cancer-related self-reported and objective cognitive function. In the broader CRCI literature, objective and subjective cognitive measures rarely correlate (1, 29, 30), so our findings suggest that serial mobile cognitive assessments in natural environments may be sensitive to both objective and subjective CRCI for breast cancer survivors.

To our knowledge, this is the largest sample used to evaluate a commercially available cognitive EMA protocol for CRCI assessment in a representative sample of breast cancer survivors, enhancing generalizability of our findings. Although recommendations about adequate sample sizes for psychometric studies vary depending on design, analytic plan, and population, most converge on a recommendation that at least 100 participants will produce adequate and reliable results for analyses focused on test–retest reliability and convergent validity (31, 32). There are several limitations to note. We did not include a matched control group, limiting our interpretation of the cognitive EMA mean scores and variability in this population, in addition to the practice effects. Consistent with our prior research, practice effects on the mobile tests were observed. When thinking about the implications of practice effects for assessing changes in patients’ cognitive profiles, it is essential to recognize that while such effects can indicate learning in cognitively unimpaired individuals, they may obscure genuine cognitive changes in clinical assessments. Future work utilizing mobile cognitive testing to understand longitudinal cognitive change in BCS needs to have an understanding of practice effects so as to ensure accurate interpretation of cognitive test results and to differentiate between test familiarity and real cognitive change*.*

Our convenience sampling methods may also represent a biased sample of survivors who are more willing to engage with smartphone research protocols. Future cognitive EMA studies should include a matched control group to facilitate interpretation of the cognitive EMA mean scores and variability in this population. We did not account for time of day/diurnal effects for EMA administration in our analyses, but did find some evidence that time of day may impact reaction time and memory scores in this population. These reaction time differences are consistent with studies examining diurnal effects of reaction time/processing speed in other populations (33, 34)*.* While it is not expected that this within-person effect would change the feasibility or psychometric findings, future studies that include hypothesis testing of cognitive EMAs in breast cancer survivors should consider this covariate. It is also possible that mood-related factors may have correlated with adherence rates in this study; however, mood-related EMAs were not accounted for in these analyses, and all participants in the “low adherence group” in this study had adherence rates over 40%, which is above the threshold for inclusion in clinical trials (35). Further, prior work by our group has found that adherence to EMA protocols is unrelated to mood symptoms [e.g., (24)].

CRCI are often mis- and under-diagnosed in clinical practice. More sensitive and ecological assessments are needed to accurately detect and manage CRCI to improve clinical outcomes for patients and survivors. These finding support previous reports that cognitive EMAs are feasible, acceptable, psychometrically sound, and potentially more sensitive than clinical cognitive assessments (13). Cognitive EMAs could be integrated into clinical settings to improve CRCI screening and treatment. Despite limitations, our findings support the feasibility, acceptability, and preliminary psychometric properties of eight commercially available, repeatable, brief cognitive EMAs for CRCI assessment in breast cancer survivors. This study contributes to a growing body of literature successfully using EMAs to assess cognitive variability in clinical populations, including cancer patients and survivors.

## Data Availability

De-identified data will be made available upon reasonable requests to the corresponding author and with data use agreements in place.
