# Supplementary material for: Feasibility, reliability and validity of smartphone administered cognitive ecological momentary assessments in breast cancer survivors
Source: Front Digit Health. 2025 Apr 22;7:1543846. doi: 10.3389/fdgth.2025.1543846 (PMC12052553; doi:10.3389/fdgth.2025.1543846)
Supplement: Supplementary file 1 [file Datasheet1.pdf]

Supplementary Table 1. Mobile cognitive testing administration schedule, every other day for 56-days								
Assessment day	N-back	Copykat	Color Trick	Matching Pair	Hand Swype	Quick Tap	Memory List	Memory Matrix
1	X	—	X[1]	X	—	—	X[3]	—
2	—	X	—	—	X	X	—	X
3	X	—	X[2]	—	—	X	X[3]	—
4	X	—	—	X	X	—	—	X
5	—	X	X[1]	X	—	—	X[4]	—
6	—	X	—	—	X	X	X[3]	—
7	X	—	—	X	X	—	—	X
8	—	X	X[2]	—	—	X	—	X
9	X	—	X[1]	—	—	X	—	X
10	X	—	X[2]	X	—	—	X[3]	—
11	X	—	—	X	X	—	X[4]	—
12	—	X	—	X	X	—	—	X
13	—	X	X[1]	—	—	X	X[3]	—
14	X	—	—	X	X	—	—	X
15	—	X	X[2]	—	—	X	—	X
16	—	X	X[1]	—	—	X	X[4]	—
17	—	X	—	X	X	—	—	X
18	X	—	—	—	X	X	—	X
19	—	X	—	X	X	—	X[3]	—
20	X	—	X[2]	—	—	X	X[4]	—
21	X	—	—	—	X	X	—	X
22	—	X	X[1]	X	—	—	X[3]	—
23	—	X	X[2]	X	—	—	—	X
24	X	—	—	—	X	X	—	X
25	—	X	X[1]	X	—	—	X[3]	—
26	X	—	X[2]	—	—	X	—	X
27	X	—	—	—	X	X	X[4]	—
28	—	X	—	X	X	—	X[3]	—
TOTAL	14	14	14[5]	14	14	14	14[6]	14

Footnote:

1: Color Trick color-to-meaning version

2: Color Trick yes/no version

3: Memory List 12 words

4: Memory List 18 words

5: 7 color to meaning version, 7 yes/no version

6: 9 administrations of the 12-word list, 5 administrations of the 18 word list

Supplementary Table 2. Cognitive EMA, Mobile Cognitive Test Specific Criteria for Outlier Removal and Number of Instances Removed

NeuroUX Cognitive Test	Outlier Removal Criteria	Number of Instances Removed
2-back	• Remove 0 values<br>• Remove person-specific outliers (>3 SD person specific mean)	• 4<br>• 7
Color Trick Scores and Reaction Time (ms)	• Remove scores & RTs from trials with scores ≤3 SD<br>• Remove scores & RTs from trials with RTs >10000ms (1 additional instance removed)<br>• Remove person-specific outliers based on 3SDs from mean cutoff	• 3 instances removed from 2 unique participants<br>• 1<br>• 5
Matching Pair Scores	Remove scores < 100	3
Memory Matrix Scores	Remove scores < 15 (this corresponds to getting fewer than 5 correct trials)	4 (from 3 unique participants)
Memory List 12 Scores	Remove scores < 12 (corresponds with a score below chance)	1
Memory List 18 Scores	Remove scores < 18 (corresponds with a score below chance)	2
CopyKat Scores	Remove scores < 4	12 instances removed from 10 unique participants
Hand Swype Scores & Reaction Time (ms)	Remove person-specific outliers based on 3SDs from mean cutoff	1
Quick Tap 1 scores	Remove person-specific outliers based on 3SDs from mean cutoff	1

Quick Tap 1 Reaction Time (ms)	Remove RTs associated with scores < 8	2
Abbreviations: ms: millisecond; RT: reaction time; SD: standard deviation		

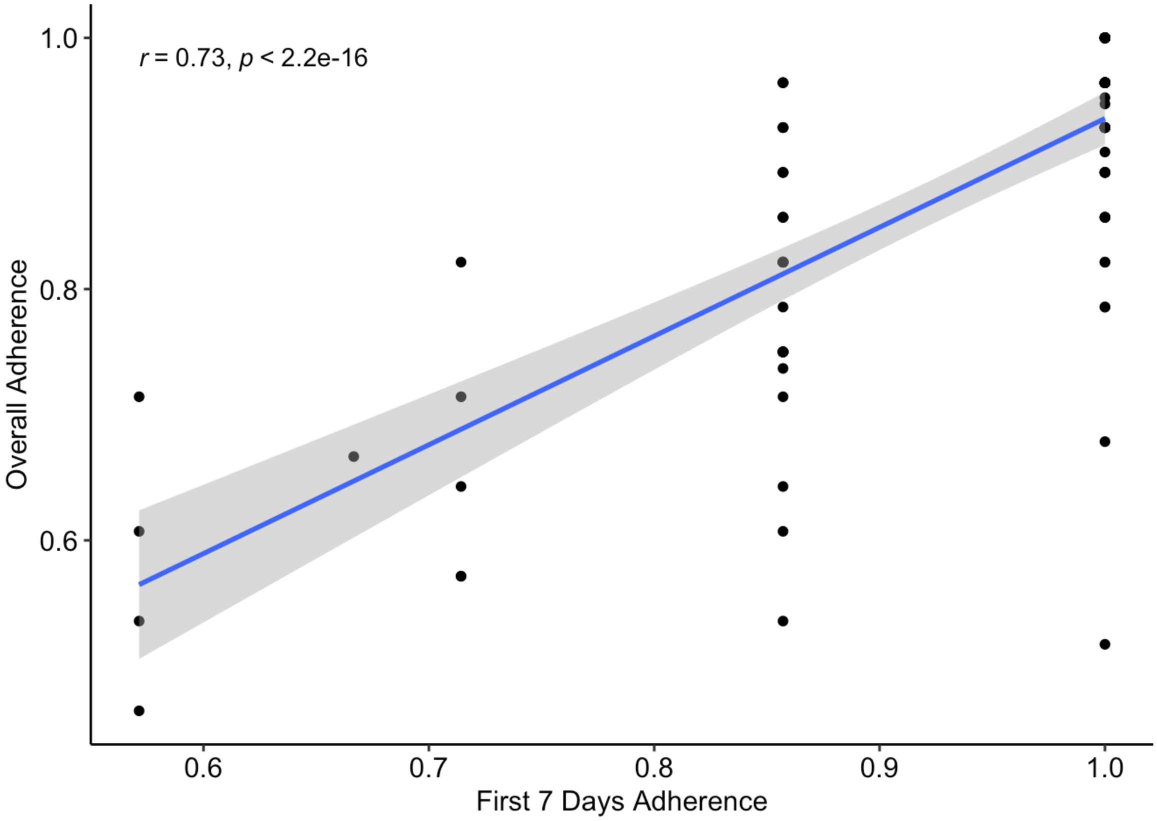

Supplementary Figure 1. Visualization of positive relationship ($r = 0.73$, $p <.0001$) between adherence in the first 7 days of the EMA protocol and overall adherence to the EMA protocol.

Supplementary Table 3. Sociodemographic, clinical, and cognitive differences in participants with low adherence compared to high adherence			
	Adherence Group		
	<70% (n=11)	>=70% (n=94)	p
Sociodemographic Factors			
Age (mean (SD))	46.09 (8.75)	51.77 (12.25)	0.139
Years of Education (mean (SD))	17.09 (1.7)	17.30 (3.01)	0.824
Hispanic ethnicity (n)	1	10	0.816
Black race (n)	0	9	0.283
Asian race (n)	0	10	0.255
White race (n)	10	68	0.182
Employed (n)	8	62	0652
Partnered	7	69	0.455
<$100K annual house hold income	4	28	0.689
Clinical Factors			
Years since treatment (mean (SD))	1.29 (0.93)	2.23 (1.67)	0.06
Breast Cancer Stage (n)			0.244
0	2	7	-
I	1	35	-
II	6	31	-
III	2	14	-
Unsure	0	5	-
History of surgery	11	92	0.625
History of radiation	7	75	0.220
History of chemotherapy	7	64	0.765
History of hormone therapy	7	57	0.847
NeuroUX EMA Self-Report Items			
crci_sx_mean (mean (SD))	2.75 (1.33)	1.52 (1.51)	0.011
cog_abilities_mean (mean (SD))	3.39 (1.15)	4.51 (1.44)	0.015
NeuroUX Mobile Cognitive Testing			
X2.back_score_mean (mean (SD))	5.72 (1.42)	6.20 (1.54)	0.333
color.trick_median.reaction.time_mean (mean (SD))	2331.00 (586.16)	2192.63 (761.02)	0.562
matching.pair_score_mean (mean (SD))	299.05 (49.10)	312.54 (60.87)	0.481
memory.matrix_score_mean (mean (SD))	43.71 (7.44)	42.84 (7.79)	0.727
memory.list.12_correct.count_mean (mean (SD))	22.26 (0.85)	21.82 (1.48)	0.333
memory.list.18_correct.count_mean (mean (SD))	30.95 (2.68)	30.95 (2.43)	0.998

copykat_score_mean (mean (SD))	10.99 (1.32)	11.55 (2.94)	0.536
Independent t tests or chi square tests used.			
hand.swype_median.reaction.time_mean (mean (SD))	1786.12 (234.19)	1809.73 (458.19)	0.873
hand.swype_score_mean (mean (SD))	27.59 (4.69)	28.59 (7.18)	0.667
quick.tap.level.1_median.reaction.time_mean (mean (SD))	408.23 (66.35)	403.40 (77.99)	0.844
BrainCheck Baseline Testing			
raw_trails_a_duration_median (mean (SD))	1.14 (0.28)	1.11 (0.28)	0.721
raw_trails_b_duration_median (mean (SD))	1.63 (0.47)	1.57 (0.41)	0.689
raw_stroop_reaction_time_median (mean (SD))	2.13 (0.37)	2.10 (0.44)	0.864
raw_digit_symbol_correct_per_second_mean (mean (SD))	0.45 (0.06)	0.43 (0.10)	0.431
raw_digit_symbol_duration_median (mean (SD))	2.14 (0.22)	2.70 (3.17)	0.577
raw_immediate_recall_correct (mean (SD))	18.50 (1.27)	19.15 (1.17)	0.102
raw_delayed_recall_correct (mean (SD))	18.50 (1.35)	18.72 (1.41)	0.634
raw_clinician_score (mean (SD))	78.35 (4.29)	78.79 (5.15)	0.794
FACT-Cog PCI			
factcogpci_total (mean (SD))	40.45 (19.74)	54.11 (17.93)	0.02

Supplementary Table 4 (See separate excel sheet)

Supplementary Table 5. Linear regression models showing associations between EMA-reported CRCI Symptoms and select cognitive performances, covarying for age			
	Outcomes		
Predictors	Baseline BrainCheck Stroop Reaction Time	Baseline BrainCheck Digit Symbol correct/second	Mean NeuroUX Memory List 18 score
Mean EMA-reported CRCI Symptoms	-0.041 (0.027)	0.007 (0.006)	-0.597 (0.158)***
Age	0.013 (0.003)***	-0.004 (0.001)***	-0.046 (0.020)*
Footnote. Values represent unstandardized beta (SE). *p<0.05, **p<0.01, ***p<0.001			

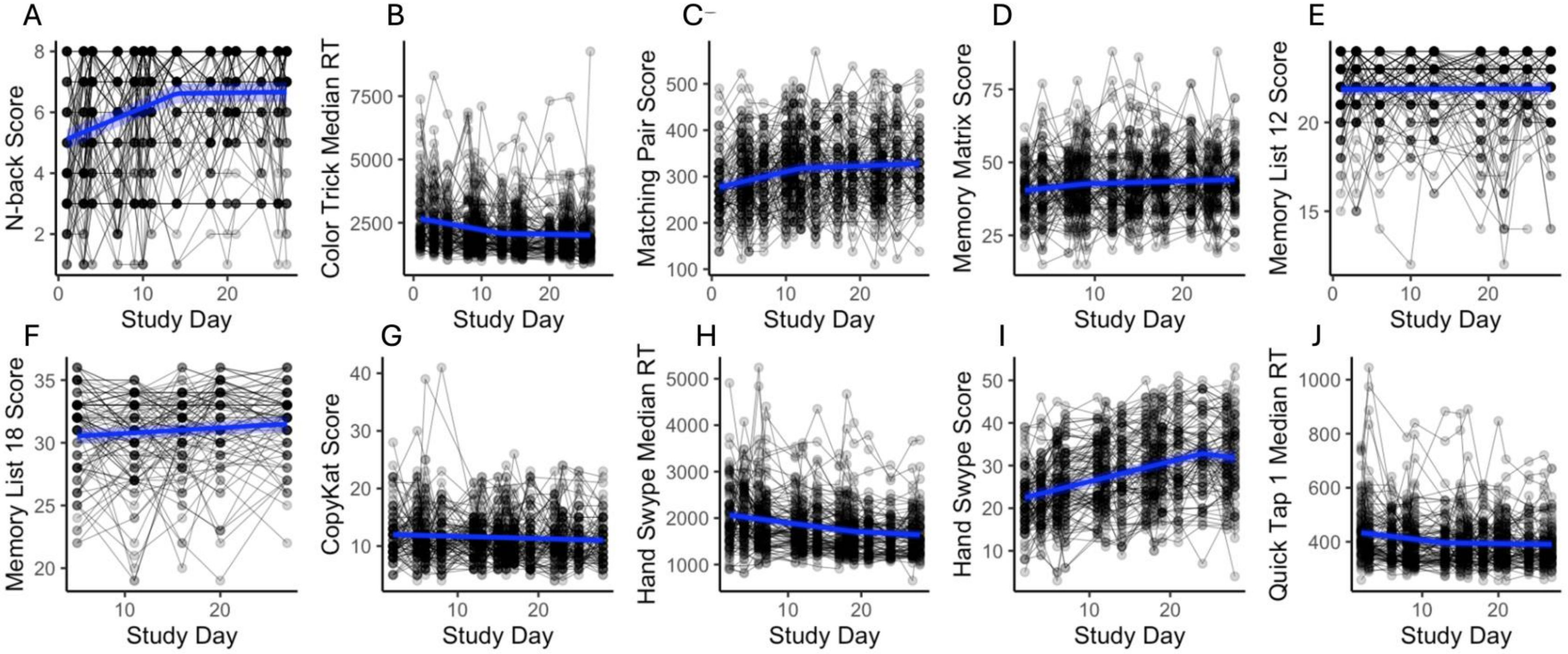

Supplementary Figure 2. Linear or nonlinear (spline) models for each cognitive test score. A) N-back score, linear splines suggest that N-back practice effects leveled off after study day 14 (days 1-14: *b* = 0.12, *SE* = 0.01, *p* < 0.001; days 15-28: *b* = 0.00, *SE* = 0.01, *p* = 0.72); B) Color Trick Median reaction time, linear splines suggest that Color Trick practice effects leveled off after study day 13 (days 1-13: *b* = -48.60, *SE* = 6.45, *p* < 0.001; days 14-28: *b* = -4.72, *SE* = 5.61, *p* = 0.40); C) Matching Pair score, linear splines suggest that Matching Pair practice effects leveled off after study day 12 (days 1-12: *b* = 3.69, *SE* = 0.49, *p* < 0.001; days 13-28: *b* = 0.63, *SE* = 0.33, *p* = 0.05); D) Memory Matrix score, linear splines suggest that Memory Matrix practice effects leveled off after study day 9 (days 1-9: *b* = 0.32, *SE* = 0.12, *p* = 0.007; days 10-28: *b* = 0.08, *SE* = 0.04, *p* = 0.06); E) Memory List 12 score, there were no consistent trends in performance over time for Memory List 12 (linear model; day: *b* = 0.001, *SE* = 0.01, *p* = 0.855); F) Memory List 18 score, there was a linear practice effect only showing that improvements in performance on Memory List 18 do not level off (day: *b* = 0.04, *SE* = 0.02, *p* = 0.01); G) CopyKat score, while there are no practice effects in CopyKat score, performance tends to decrease

over time (day: *b* = -0.04, *SE* = 0.01, *p* = 0.005); H) Hand Swype reaction time, linear splines suggest that Hand Swype Reaction time leveled off after day 19 (days 1-19: *b* = -20.80, *SE* = 2.07, *p* < 0.001; days 20-28: *b* = -7.93, *SE* = 4.30, *p* = 0.07); I) Hand Swype score, linear splines suggest that Hand Swype Score practice effects leveled off after day 24 (days 1-24: *b* = 0.47, *SE* = 0.02, *p* < 0.001; days 25-28: *b* = -0.26, *SE* = 0.14, *p* = 0.07); J) Quick Tap reaction time, linear splines suggest that Quick Tap 1 practice effects leveled off after day 13 (days 1-13: *b* = -3.39, *SE* = 0.52, *p* < 0.001; days 14-28: *b* = -0.41, *SE* = 0.42, *p* = 0.33).

Supplementary Table 6. Linear mixed effects models for time of day and EMA cognitive test administration

**Outcome**	**Predictor**	**Est.**	**S.E.**	**p**
NBack	(Intercept)	6.138	0.186	<0.001
NBack	sessiontimeofdayMid-Day (ref: Morning)	0.024	0.134	0.859
NBack	sessiontimeofdayEvening (ref: Morning)	-0.026	0.138	0.850
NBack	sessiontimeofdayNight (ref: Morning)	0.07	0.15	0.639
NBack	sessiontimeofdayLate Night (ref: Morning)	-0.273	0.485	0.574
**Outcome**	**Predictor**	**Est.**	**S.E.**	**p**
Color Trick Reaction Time	(Intercept)	2127.817	95.925	<0.001
Color Trick Reaction Time	sessiontimeofdayMid-Day (ref: Morning)	87.157	75.986	0.252
Color Trick Reaction Time	sessiontimeofdayEvening (ref: Morning)	39.023	78.109	0.617
Color Trick Reaction Time	sessiontimeofdayNight (ref: Morning)	144.263	84.487	0.088

Color Trick Reaction Time	sessiontimeofdayLate Night (ref: Morning)	580.511	248.87	**0.020**
**Outcome**	**Predictor**	**Est.**	**S.E.**	**p**
Matching Pair	(Intercept)	310.722	7.243	<0.001
Matching Pair	sessiontimeofdayMid-Day (ref: Morning)	6.854	5.29	0.195
Matching Pair	sessiontimeofdayEvening (ref: Morning)	-0.965	5.333	0.856
Matching Pair	sessiontimeofdayNight (ref: Morning)	-5.114	5.969	0.392
Matching Pair	sessiontimeofdayLate Night (ref: Morning)	-26.342	15.995	0.100
**Outcome**	**Predictor**	**Est.**	**S.E.**	**p**
Memory Matrix	(Intercept)	42.531	1.016	<0.001
Memory Matrix	sessiontimeofdayMid-Day (ref: Morning)	0.678	0.809	0.403
Memory Matrix	sessiontimeofdayEvening (ref: Morning)	0.397	0.831	0.633
Memory Matrix	sessiontimeofdayNight (ref: Morning)	-0.001	0.912	0.999
Memory Matrix	sessiontimeofdayLate Night (ref: Morning)	0.475	2.438	0.846
**Outcome**	**Predictor**	**Est.**	**S.E.**	**p**
Memory List 12	(Intercept)	21.486	0.221	<0.001
Memory List 12	sessiontimeofdayMid-Day (ref: Morning)	0.474	0.211	**0.025**
Memory List 12	sessiontimeofdayEvening (ref: Morning)	0.481	0.215	**0.026**

Memory List 12	sessiontimeofdayNight (ref: Morning)	0.285	0.235	0.225
Memory List 12	sessiontimeofdayLate Night (ref: Morning)	0.18	0.768	0.815
**Outcome**	**Predictor**	**Est.**	**S.E.**	**p**
Memory List 18	(Intercept)	31.108	0.436	<0.001
Memory List 18	sessiontimeofdayMid-Day (ref: Morning)	0.139	0.458	0.762
Memory List 18	sessiontimeofdayEvening (ref: Morning)	-0.261	0.464	0.574
Memory List 18	sessiontimeofdayNight (ref: Morning)	-0.547	0.516	0.290
Memory List 18	sessiontimeofdayLate Night (ref: Morning)	0.249	1.227	0.840
**Outcome**	**Predictor**	**Est.**	**S.E.**	**p**
CopyKat	(Intercept)	11.439	0.399	<0.001
CopyKat	sessiontimeofdayMid-Day (ref: Morning)	0.18	0.349	0.606
CopyKat	sessiontimeofdayEvening (ref: Morning)	0.117	0.352	0.740
CopyKat	sessiontimeofdayNight (ref: Morning)	-0.18	0.394	0.647
CopyKat	sessiontimeofdayLate Night (ref: Morning)	-0.781	0.965	0.419
**Outcome**	**Predictor**	**Est.**	**S.E.**	**p**
Hand Swype Reaction Time	(Intercept)	1796.887	54.253	<0.001

Hand Swype Reaction Time	sessiontimeofdayMid-Day (ref: Morning)	19.518	39.616	0.622
Hand Swype Reaction Time	sessiontimeofdayEvening (ref: Morning)	10.672	40.15	0.790
Hand Swype Reaction Time	sessiontimeofdayNight (ref: Morning)	10.636	44.773	0.812
Hand Swype Reaction Time	sessiontimeofdayLate Night (ref: Morning)	-155.258	119.541	0.194
**Outcome**	**Predictor**	**Est.**	**S.E.**	**p**
Hand Swype Score	(Intercept)	28.798	0.854	<0.001
Hand Swype Score	sessiontimeofdayMid-Day (ref: Morning)	-0.517	0.621	0.405
Hand Swype Score	sessiontimeofdayEvening (ref: Morning)	-0.096	0.629	0.879
Hand Swype Score	sessiontimeofdayNight (ref: Morning)	-0.629	0.702	0.370
Hand Swype Score	sessiontimeofdayLate Night (ref: Morning)	3.042	1.874	0.105
**Outcome**	**Predictor**	**Est.**	**S.E.**	**p**
Quick Tap 1	(Intercept)	390.637	8.981	<0.001
Quick Tap 1	sessiontimeofdayMid-Day (ref: Morning)	8.862	5.949	0.137
Quick Tap 1	sessiontimeofdayEvening (ref: Morning)	17.553	6.086	**0.004**
Quick Tap 1	sessiontimeofdayNight (ref: Morning)	21.226	6.659	**0.001**
Quick Tap 1	sessiontimeofdayLate Night (ref: Morning)	8.631	18.705	0.645

Footnote. Time of day was treated as a categorical variable, coded as follows using the participant's local time zone: Morning (0600-1100); Mid-Day (1101-1600); Early evening (1601-2000); Night (2001-2359); and Late night (2400-0559).

.
